# Drug Use during Pregnancy and its Consequences: A Nested Case Control Study on Severe Maternal Morbidity

**DOI:** 10.1055/s-0038-1667291

**Published:** 2018-07-31

**Authors:** Cynara Maria Pereira, Rodolfo Carvalho Pacagnella, Mary Angela Parpinelli, Carla Betina Andreucci, Dulce Maria Zanardi, Renato Souza, Carina Robles Angelini, Carla Silveira, José Guilherme Cecatti

**Affiliations:** 1 Universidade Estadual de Campinas, Campinas, SP, Brazil; 2 Universidade Federal de São Carlos, São Carlos, SP, Brazil

**Keywords:** drug use, illicit drugs, pregnancy, high risk pregnancy, child development, uso de drogas, drogas ilícitas, gestação, gestação de alto risco, desenvolvimento infantil

## Abstract

**Objective** To assess the relationship between the use of psychoactive substances during pregnancy and the occurrence of severe maternal morbidity (SMM), perinatal outcomes and repercussions on the neuropsychomotor development of exposed children.

**Methods** A case-control study nested within a cohort of severe maternal morbidity (COMMAG) was performed. Women with SMM were considered cases. Controls were those with low-risk pregnancy, without SMM and admitted during the same time period as the cases. Cohort data were collected retrospectively in hospital records for childbirth. A face-to-face interview was also performed with 638 women (323 without SMM and 315 with SMM) and their children of the index pregnancy between 6 months and 5 years after childbirth. During the interview, substance abuse during pregnancy was assessed by a modified question from the Alcohol, Smoking and Substance Involvement Screening Test 2.0 (ASSIST) and the neuropsychomotor development in the children was assessed by the Denver Developmental Screening Test, 2^nd^ edition.

**Results** The prevalence of licit or illicit drug use during pregnancy was ∼ 17%. Among drug users, 63.9% used alcohol, 58.3% used tobacco, 9.2% used cocaine/crack and 4.6% used marijuana. There was no association between drug use during pregnancy and SMM, although tobacco use during pregnancy was associated with bleeding, presence of near-miss clinical criteria (NMCC) and alteration in infant development; alcohol use was associated with neonatal asphyxia; and cocaine/crack use was associated with the occurrence of some clinical complications during pregnancy.

**Conclusion** The use of psychoactive substances during pregnancy is frequent and associated with worse maternal, perinatal and child development outcomes.

## Abstract (pt)

**Resumo:**

**Objetivo** Avaliar a relação entre o uso de substâncias psicoativas na gestação e a ocorrência de morbidade materna grave (MMG), resultados perinatais e repercussões no desenvolvimento neuropsicomotor das crianças expostas.

**Métodos** Estudo de caso-controle a partir de uma coorte de morbidade materna grave (COMMAG). Mulheres com MMG foram consideradas casos. Controles foram mulheres com gestação de baixo risco, admitidas no mesmo período que os casos. Os dados da coorte foram coletados retrospectivamente em prontuários de internação para o parto e entrevistas presenciais conduzidas com 638 mulheres e seus filhos da gestação-índice, entre 6 meses e 5 anos após o parto. Na entrevista, o uso de substâncias na gestação foi avaliado com uma pergunta modificada introduzida no questionário para triagem do uso de álcool, tabaco e outras substâncias 2.0 (ASSIST, na sigla em inglês) e o desenvolvimento neuropsicomotor das crianças foi avaliado pelo teste de triagem do desenvolvimento Denver II.

**Resultados** A prevalência do uso de drogas lícitas ou ilícitas na gestação foi de cerca de 17%. Das usuárias, 63,9% usaram álcool, 58,3% usaram tabaco, 9,2% usaram cocaína/crack e 4,6% usaram maconha. Não houve associação entre o uso de drogas na gestação e MMG. Contudo, o uso de tabaco foi associado a hemorragia, presença de critérios clínicos de *near miss* e alteração no desenvolvimento infantil. O uso de álcool foi associado à asfixia neonatal e o uso de cocaína/crack à ocorrência de alguma complicação clínica na gestação.

**Conclusão** O abuso de substâncias lícitas ou ilícitas na gestação é frequente e associado a piores desfechos maternos, perinatais e do desenvolvimento infantil.

## Introduction

In 2012, it was estimated that 162 to 324 million people used an illicit drug, which accounts for ∼ 3.5 to 7% of the world population aged 15 to 64 years old. In general, the most widely used illicit drugs belong to the cannabinoid, opioid, cocaine and amphetamine-type stimulant groups.^1^ In the USA, the National Survey on Drug Use and Health (NSDUH) indicated that 4.7% of pregnant women used illicit substances in 2015. Furthermore, 9.3% of these women used alcohol and 13.6% used tobacco.^2^ As a result, a large number of fetuses were exposed to these substances during the embryonic development stage. In 2012, it was estimated that ∼ 380,000 newborn infants were exposed to illicit substances in the US. Over 550,000 were exposed to alcohol and more than 1 million were exposed to tobacco before birth.^3^

During pregnancy, drug use has various potential effects on the fetus. Tobacco, the most commonly used substance, may lead to low fetal weight, growth restriction and prematurity. It can even be associated with an increased incidence of perinatal deaths.^4^ ^5^ Alcohol is a teratogenic agent and exposure to this substance is associated with effects that include prenatal or postnatal growth restriction, central nervous system dysfunction and a characteristic pattern of facial anomalies.^6^ ^7^ ^8^ Other widely used drugs are derived from cocaine and are also associated with preterm birth, low birthweight and fetuses that are small for gestational age (SGA).^9^ Marijuana, amphetamines and opioids are other drugs used.^3^ ^10^

Although the fetal consequences of drug use have been well studied, problems are not limited to the fetuses. Maternal and familial consequences may be as severe or worse than the fetal problems.^11^ ^12^ Users of psychoactive substances have a lower number of medical consultations or fail to receive prenatal follow-up care. These women have a worse global and mental health before pregnancy, low socioeconomic level and inadequate nutritional levels.^3^ ^13^ ^14^

Substance abuse during pregnancy may lead to increased rates of sexually transmitted diseases and HIV infection in addition to pathologic conditions during pregnancy, such as hemorrhages, especially abruptio placenta, hypertensive crisis and myocardial infarction.^15^ These same conditions are frequently associated with severe maternal morbidity (SMM) and death.

In Brazil, as well as in other countries, the major conditions associated with maternal morbimortality are related to bleeding and hypertension.^16^ In both conditions, drug use is a major risk factor.^15^ However, available information on the use of illicit substances among Brazilian pregnant women, and its association with adverse maternal/perinatal results is scarce.

Due to the increasing number of drug users in the reproductive-aged female population and with the purpose of improving maternal and fetal health, it is crucial to understand the maternal and fetal repercussions of substance abuse during pregnancy for the recognition and modification of the problem.^17^ The aim of the current study was to evaluate a possible association between SMM and licit and/or illicit substance abuse during pregnancy. Another aim of the current study was to trace a risk profile for maternal morbidity in pregnant drug users, evaluating perinatal results and the repercussions on infant development.

## Methods

The present analysis used a case-control strategy, nested in a retrospective cohort study of SMM with a fixed comparison group, the COMMAG. This cohort was planned to study the lifetime effect of SMM on women, compared with a group of women without maternal morbidity. The presence of SMM was the originally studied exposure. Details on the methods of the present study have already been previously published.^18^ Briefly, this cohort was developed at the Centro de Atenção Integral à Saúde da Mulher (Casim, in the Portuguese acronym), at Univerisdade Estadual de Campinas (UNICAMP, in the Portuguese acronym), Brazil, to conduct a multidimensional study on the pospartum repercussions of SMM in a period ranging from 1 to 5 years after chidlbirth. The mother and child (born in the index pregnancy) were evaluated in relation to several aspects by means of standardized questionnaires. Personal data, reproductive history, sociodemographic and general health profiles as well as perinatal results (gestational age; appearance, pulse, grimace, activity, respiration [Apgar] score; and perinatal outcome) were collected. Prevalidated questionnaires were also applied to assess quality of life (Short Form Health Survey 36 [SF-36]), posttraumatic stress disorder (Posttraumatic Stress Disorder Checklist – Civilian Version [PCL-C]), daily functioning (World Health Organization's Disability Assessment Schedule 2.0 [WHODAS II]), and sexual functioning (Female Sexual Function Index [FSFI]), in addition to the Alcohol, Smoking and Substance Involvement Screening Test 2.0 (ASSIST) for the assessment of substance use.

The children were evaluated according to the World Health Organization's (WHO) weight-height curves and by the Denver Developmental Screening Test, 2^nd^ edition. The Denver test screens asymptomatic children and assesses language, fine and gross motor skills and personal-social interaction; at the end, the test offers an initial evaluation of potential neuropsychomotor developmental delay.

The study population was composed of women admitted to the institute from January 2008 to July 2012, presenting or not any SMM condition during pregnancy, delivery and postpartum period. During this period, 1,157 women who met the selection criteria for the study were retrospectively identified, including all women with SMM and the same number of women without SMM, randomly selected (balanced per year of delivery). The women were contacted by telephone during a period that ranged from 6 months to 5 years after childbirth.

For each medical chart selected, three attempts were made to contact each woman. In the case of unsuccessful attempts, an invitation letter for participation in the present study was sent to the women. The telephone number of the research center was included in the letter to provide contact with the researchers and formalize the study.

On this first phone contact, an interviewer especially trained in teleresearch informed the woman about the study objectives, inviting her to participate in the study. A consent term was read to those agreeing to participate and recorded along with the woman's verbal acceptance. At the same time, the woman responded to a series of questions about sociodemographic characteristics, general health and reproductive history, as well as questionnaires on quality of life (SF-36) and posttraumatic stress disorder (PCL-C). After the first contact, the woman was invited to continue in the study. She was required to participate in a face-to-face interview in the healthcare service scheduled at her convenience, along with the child corresponding to the pregnancy assessed in the present study. On the second contact, the woman was interviewed by a trained multiprofessional team and responded to standardized questionnaires for sexual functioning (FSFI), functioning (WHODAS II) and substance use (ASSIST 2.0). In addition, a trained pediatrician evaluated the child's growth and development using the Denver scale, 2^nd^ edition.

A case-control strategy was used for the current analysis. The diagnosis of SMM was based on the presence of potentially life-threatening conditions and/or maternal near-miss events, according to criteria defined by the WHO.^19^ These conditions did not cause death by chance or medical care and led to admission in an intensive care unit (ICU). The control group consisted of women who had not experienced this condition and was composed of women discharged from the rooming-in unit during the same period, after giving birth to a live newborn infant without malformations at more than 37 weeks of gestation (calculated by the date of the last menstruation or first semester ultrasound and confirmed by somatic age assessment of the newborn infant using the Capurro method).

The main risk factor evaluated in the present analysis was drug use during the index pregnancy according the women's personal declaration of using any psychoactive substance. To address this issue, a modified question was used from the ASSIST 2.0, an instrument that had already been translated and validated to the Portuguese language.^20^ This question sought information about the use of psychoactive substance during the index pregnancy and could elicit the following responses: no drugs, use of cigarette or derivatives of tobacco, alcoholic beverages, marijuana, cocaine/crack, amphetamines or ecstasy, hypnotic/sedative inhalants, hallucinogens, opioids and others.

The ASSIST 2.0 was developed by the WHO.^21^ It provides the profile of lifetime and previous 3-month nonmedical substance use. The first question is about lifetime substance use, including the school period. For each substance declared, question 2 assesses the frequency of drug use in the past 3 months. At the end, the weighted sum response to questions 2 to 7, for each drug declared, provides the pattern for the need to intervene or not. In scores ranging from 0–3 = no intervention, 4–26= brief intervention and 27 or higher = patient referral for a more intensive care. The assessment of drug use in the previous 3 months, however, was not the object of the current study.

## Sample Size

For a complete cohort, the sample size was defined by the need to identify differences in quality of life. For this purpose, 337 women were required per group, in a total of 674 participants. To evaluate postpartum drug use, the prevalence of drug use during pregnancy was considered to be 15%.^3^ The prevalence of concomitant drug use with a severe morbid condition was 34%.^22^ Therefore, using type I error of 1% and type II error of 10%, the number required to find this difference would be 322 subjects (161 per group).

## Analysis

To initially evaluate the sample profile according to sociodemographic, obstetric and neonatal characteristics, frequency tables of diverse categories of qualitative variables with absolute (*n*) and percentage (%) frequency values were constructed, comparing cases to controls. The difference between these groups was evaluated by the chi-squared test, estimating odds ratio (OR) with its respective 95% confidence interval (CI). The prevalence of drug use during pregnancy was then descriptively evaluated.

To study the association between the use of distinct drug categories and specific SMM conditions, the use of diverse drugs was comparatively evaluated between groups by the chi-squared test and an estimate of the OR and 95% CI. Then, neonatal outcomes (prematurity, vitality at birth) and neuropsychomotor development of the child at the time of the interview were assessed with respect to drug use by the chi-squared test, estimating the respective OR and 95% CI. The level of significance adopted for the study was 5%.

## Results

**Fig. 1:**
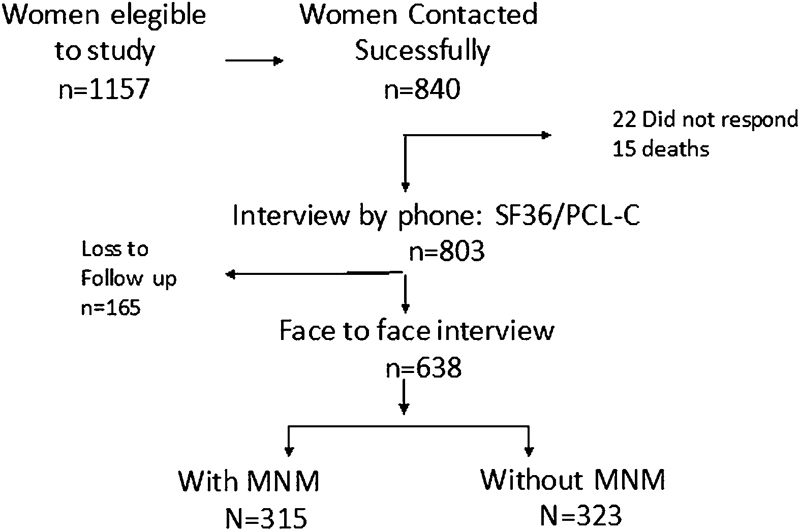
Flow chart of women's participation in the study.Abbreviations: MNM, Maternal Near Miss.

At the end of the second phase of the present study, which was the face-to-face interview, there were 638 interviewees (323 controls, 315 cases). Of the 315 cases, 248 women had potentially life-threatening conditions and 67 had near-miss events (Fig. 1). Table 1 describes the sociodemographic, obstetric and neonatal variables in both sample groups.

**Table 1:** Sociodemographic, obstetric and neonatal characteristics in women with and without severe maternal morbidity

Characteristics	With SMM	Without SMM	OR (95% CI)	*p*-value*
	*n* (%)	*n* (%)		
Age				
≤ 19 years old	10 (3.1)	19 (5.8)	0.65 (0.29–1.43)	0.001
20–34 years old	189 (60)	234 (72.4)	ref	
≥ 35 years old	116 (36)	70 (21.6)	3.14 (1.38–7.15)	
Color				
White	152 (48.2)	133 (41.1)	ref	0.07
Non-white	163 (51.7)	190 (58.8)	0.75 (0.54–1.02)	
Marital status^a^				
Lives together	257 (81.5)	268 (83.2)	ref	0.58
Does not live together	58 (18.4)	54 (16.7)	1.12 (0.74–1.68)	
Religious				
Yes	288 (91.4)	277 (85.7)	ref	0.02
No	27 (8.5)	46 (14.2)	0.56 (0.34–0.93)	
School Education^b^				
< 8 years	141 (45.1)	133 (41.1)	1.23 (0.71–2.12)	0.58
8 - 11 years	141 (45.1)	155 (48)	1.06 (0.61–1.81)	
> 11 years	30 (9.6)	35 (10.8)	Ref	
Economic class^c^				
A + B	117 (37.7)	140 (43.3)	Ref	0.15
C + D + E	193 (62.2)	183 (56.6)	1.26 (0.91–1.73)	
Number of pregnancies				
1	102 (32.3)	109 (33.7)	ref	0.60
2	93 (29.5)	103 (31.8)	0.96 (0.65–1.42)	
≥ 3	120 (38.1)	111 (34.3)	1.19 (0.81–1.75)	
GA at birth				
< 37	154 (49.5)	36 (11.1)	7.82 (5.18–11.80)	< 0.001
≥ 37	157 (50.4)	287 (88.8)	ref	
Apgar score at 5 minutes ^d^				
< 7	28 (9.3)	8 (2.4)	4.52 (1.67–12.2)	0.002
≥ 7	273 (90.7)	314 (97.5)	ref	
Perinatal outcome ^e^				
Live	301 (96.4)	322 (99.7)	ref	< 0.018
Stillborn	11 (3.53)	1 (0.31)	11.76 (1.51–91.68)	

Abbreviations: Apgar, appearance, pulse, grimace, activity, respiration; CI, confidence interval; GA, gestational age; OR, odds ratio; SMM, severe maternal morbidity; ref, reference standards.

* Chi-squared. Missing: a - 1, b - 3, c - 5, d - 15, e- 3.

The sample groups were basically homogeneous, differing in age and religion. Women aged ≥ 35 years old have a 3-fold higher estimated risk for SMM (OR = 3.14; CI = 1.38–7.15), and women who declared to have a religion had a 77% higher risk of SMM (OR = 1.77; CI = 1.07–2.92).

The sample groups were similar in terms of the number of pregnancies. However, all the remaining obstetric and neonatal variables show that the SMM group had the worst obstetric results. In the SMM group, ∼ 50% of the deliveries occurred at less than 37 weeks of gestation (OR = 7.82; CI = 5.18–11.80). There was also a 4-fold increased rate of Apgar score < 7 at 5 minutes (OR = 4.02; CI = 1.80–8.98), and 3.5% of stillborns compared with 0.3% in the group without SMM. Finally, giving birth by cesarean route was associated with a 5-fold higher estimated risk for SMM (OR = 5.25; CI = 3.65–7.56).

The use of any drug during pregnancy was reported by 16.9% of women. Alcohol was used by 10.8%, and tobacco by 9.9% of women. Of those using any drug, 63.9% used alcohol, 58.3% used tobacco, 4.6% used marijuana, 9.2% used cocaine/crack and only 0.9% used inhalants. Other drugs (opioids, amphetamines, hypnotics and hallucinogens) were not reported by any woman interviewed. Drug association was uncommon; of the woman who used drugs during pregnancy, 71% used only one drug. However, 28.6% declared a concomitant use of more than one drug (23.1% used two drugs, 5.5% used 3 or more drugs) (data not shown in Tables).

Evaluating drug use in relation to the maternal morbidity group, no significant differences were observed. Drug use during pregnancy (tobacco, alcohol and cocaine/crack) was not associated with severe maternal morbidity (Table 2).

**Table 2:** Drug use during pregnancy in women with and without severe maternal morbidity

Drug use	With SMM	Without SMM	Total	
Type of drug	*n* (%)	*n* (%)	*n* (%)	*p*-value*
No use	261 (82.9)	268 (83.0)	530 (83.1)	0.96
Tobacco	34 (10.8)	29 (9.0)	63 (9.9)	0.44
Alcohol	34 (10,8)	35 (10.8)	69 (10.8)	0.98
Cocaine	7 (2.2)	03 (0.9)	10 (1.6)	0.20
Any drug	54 (17.1)	54 (16.7)	108 (16.9)	0.88

Abbreviation: SMM, severe maternal morbidity.

*Chi-Squared: The sum of tobacco, alcohol and cocaine use is greater than the total use of any individual drug, due to concomitant drug use. Marijuana use is not present on the tables because it was not possible to perform the analysis due to the limited number of users in the sample.

When different causes of maternal morbidity were evaluated, however, tobacco use during pregnancy increased by ∼ 2-fold the risk of SMM due to bleeding in the presence of near-miss clinical criteria (NMCC). In contrast, cocaine use increased by 5-fold the estimated risk of occurrence of pregnancy-related clinical complications (Table 3).

**Table 3:** Association between drug use during pregnancy and specific severe maternal morbid conditions (hemorrhage, hypertension, other complication and NMCC)

Drug use	With hemorrhage	Without hemorrhage	*p*-value	OR	95% CI
Type of drug	*n* (%)	*n* (%)			
No use	44 (78.6)	485 (83.3)	0.34	1.0	–
Tobacco	10 (17.9)	53 (9.1)	0.04	2.17	1.03–4.54
Alcohol	6 (10.7)	63 (10.8)	0.97	0.98	0.40–2.39
Cocaine	2 (3.6)	8 (1.4)	0.22	2.65	0.55–12.8
Any drug	12 (21.4)	96 (16.5)	0.34	1.38	0.70–2.71
	**With hypertension**	**Without hypertension**			
**Type of drug**	**n (%)**	***n*** **(%)**	***p*** **-value**	**OR**	**95% CI**
No use	158 (83.6)	371 (82.6)	0.76	1.0	–
Tobacco	20 (10.6)	43 (9.6)	0.69	1.11	0.63–1.95
Alcohol	22 (11.6)	47 (10.5)	0.66	1.12	0.65–1.92
Cocaine	3 (1.6)	7 (1.6)	0.97	1.01	0.26–3.98
Any drug	31 (16,4)	77 (17.1)	0.81	0.94	0.60–1.49
**Type of drug**	**With another complication**	**Without another complication**	***p*** **-value**	**OR**	**95% CI**
	***n*** ** (%)**	***n*** **(%)**			
No use	86 (78.9)	443 (83.7)	0.22	1.0	–
Tobacco	15 (13.8)	48 (9.1)	0.13	1.59	0.86–2.97
Alcohol	12 (11)	57 (10.8)	0.94	1.02	0.53–1.98
Cocaine	5 (4.6)	5 (0.9)	0.01	5.00	1.43–17.7
Any drug	23 (21.1)	85 (16.1)	0.20	1.39	0.83–2.33
**Type of drug**	**With NMCC**	**Without NMCC**	***p*** **-value**	**OR**	**95% CI**
	***n*** **(%)**	***n*** **(%)**			
No use	24 (72.7)	505 (83.5)	0.11	1.0	–
Tobacco	7 (21.2)	56 (9.3)	0.03	2.63	1.09–6.35
Alcohol	3 (9.1)	66 (10.9)	0.74	0.81	0.24–2.75
Cocaine	1 (3.0)	9 (1.5)	0.49	1.0	–
Any drug	9 (27.3)	99 (16.4)	0.10	1.91	0.86–4.24

Abbreviations: CI, confidence interval; NMCC, near miss clinical criteria; OR, odds ratio.

Other pregnancy-related complications included the presence of any of the following conditions: pulmonary edema, seizures, thrombocytopenia < 100,000, thyroid storm, shock, acute respiratory failure, acidosis, cardiopathy, stroke, coagulation disorder, disseminated intravascular coagulation (DIC), thromboembolism, diabetic ketoacidosis, jaundice/liver dysfunction, meningitis, severe sepsis, acute renal failure (ARF). The NMCC were cyanosis, cerebrovascular disease (stroke), gasping, uncontrolled seizure/total paralysis, respiratory rate (RR) > 40 or < 6, jaundice in the presence of preeclampsia, shock, oliguria that does not respond to fluids or diuretics, coagulation disorder, loss of consciousness for 12 hours or more, unconsciousness and absence of pulse/heart beat. The use of marijuana is not present in the tables because it was not possible to perform an analysis due to the limited number of users in the sample.

Concerning neonatal outcome, drug use was not associated with prematurity. Nevertheless, it increased by 2.3 times the risk of an Apgar score < 7 in newborns. Alcohol use showed the greatest risk for this outcome (Table 4).

**Table 4:** Association between drug use during pregnancy and some neonatal results and child development

Drug use *^a^	Prematurity ^a^		*p*-value	OR	95% CI
	≤ 36 weeks	≥ 37 weeks			
	*n* (%)	*n* (%)			
No	154 (81.1)	372 (83.8)	0.35	1.0	−
**Yes**	36 (18.9)	72 (16.2)		1.26	0.77–2.07
Type of drug*^b^	**Apgar score**		***p*** **-value**	**OR**	**95% CI**
	**≤ 6**	**≥ 7**			
	**n (%)**	**n (%)**			
Any drug	9 (36)	97 (16.2)	0.013	2.93	1.24–6.90
Tobacco	5 (20)	56 (9.4)	0.11	2.31	0.82–6.49
Alcohol	7 (28)	61 (10.2)	0.008	3.51	1.38–8.90
Cocaine	0	9 (1.5)	0.98	1.0	−
Type of drug*^c^	**Neuropsychomotor development—Denver Scale, 2^nd^ edition**		***p*** **-value**	**OR**	**95% CI**
	**Suspicion of delay**	**No delay**			
	***n*** **(%)**	***n*** **(%)**			
Any drug	15 (19.7)	79 (16)	0.42	1.2	0.69–2.38
Tobacco	12 (15.8)	41 (8.3)	0.04	2.0	1.03–4.06
Alcohol	5 (6.6)	56 (11.4)	0.20	0.54	0.20–1.40
Cocaine	4 (5.3)	4 (0.8)	0.01	5.9	1.45–24.3

Abbreviations: Apgar, appearance, pulse, grimace, activity, respiration; CI, confidence interval; OR, odds ratio.

*Missing: a = 4; b = 15, c = 83. Marijuana use is not present on the tables because it was not possible to perform the analysis due to the limited number of users in the sample.

The use of tobacco and cocaine was associated with child developmental delay in the Denver scale, 2^nd^ edition, from a period of 6 months to 5 years after delivery.

Finally, a multiple analysis for the evaluation of factors related to maternal morbidity considered sociodemographic/gestational variables and drug use. Drug use was not shown to be a factor associated with general maternal morbidity. Factors independently associated with maternal morbidity were gestational age lower than 37 weeks, cesarean delivery, Apgar score < 7 at 5 minutes and low social level (data not shown in the Tables).

## Discussion

In the current study, we found that the prevalence of licit or illicit drug use during pregnancy was 16.9%. Among users of any substance, it was shown that 28.6% of women combined the use of 2 or more drugs. This value was slightly lower than the number found in the international literature, but very close to the Brazilian data. In the US, the NSDUH from 2015 revealed that 21.7% of the pregnant women used illicit substances, tobacco or alcohol during pregnancy, and that multiple drug use was common.^2^ In Brazil, a cross-sectional study conducted with 395 pregnant women showed a prevalence of 18.2% for licit or illicit substance abuse, with 9.1% for tobacco, 6.0% for alcohol and 1.0% for illicit substances (crack and marijuana).^23^

This difference was probably due to the characteristics of the study, which was not planned to analyze prevalence. Nevertheless, these numbers are worrisome and in fact may be even higher. Some women omit to declare substance abuse during pregnancy because they may feel uncomfortable.^13^ However, the study indicated that licit or illicit abuse of psychoactive substances during pregnancy is a reality in Brazil.^3^ Furthermore, there is evidence that substance abuse has several undesirable consequences on the mother and the fetus.^15^ ^24^ ^25^ ^26^

Concerning the consequences of drug use on the pregnant woman and the fetus, when SMM is considered as a whole, no differences in licit or illicit drug use during pregnancy were identified in the groups with and without SMM. No other comparative study exists in the literature, since a previous evaluation of the consequences of drug use was not conducted with a composite outcome using SMM, as we have done. However, when we evaluated each drug separately in relation to the different main determinants of SMM, some relevant results were observed.

In tobacco users, we have identified a 2-fold increased risk of hemorrhage, which is in agreement with the data in the literature. It is well documented that smokers have an increased risk of presenting with placenta previa and abruptio placenta, premature labor, fetuses with low weight and premature rupture of membranes.^5^ ^27^ Furthermore, in our study, the NMCC were twice more common among smokers. These criteria include the presence of pathologic conditions such as stroke and coagulation disorders, which are potentially linked to tobacco use and may be potentiated by pregnancy.^28^ ^29^

In cocaine users, the risk of diagnosing other complications, such as pulmonary edema, thrombocytopenia, seizures, shock, and respiratory failure, among others, was five-fold higher. This is in agreement with the literature, which shows that these complications are caused by drug effects on the organism, potentiating adrenergic effects. During pregnancy, the effects are even greater due to modifications in the metabolism.^30^

Concerning the perinatal results, we have found that the use of any drug doubled the estimated risk of a low Apgar score at 5 minutes of life. Drug stratification showed that alcohol was the main agent responsible for this outcome. A similar result was described by a Swiss study that evaluated alcohol consumption in more than 1,000 thousand women through questionnaires. It was concluded that moderate to high alcohol consumption was correlated with neonatal asphyxia.^31^

Regarding infant development within 5 years after chidbirth, the result of infant assessment by the Denver scale, 2^nd^ edition, showed that children of women who smoked tobacco during pregnancy had virtually twice the alteration in the Denver test, especially changes detected in language. A recent review on the topic concluded that there is weak evidence suggesting that active or passive maternal exposure to tobacco and small to moderate alcohol consumption during pregnancy may affect infant neuropsychomotor development.^32^ However, this evaluation is difficult to carry out due to confounding variables in the samples, such as the concomitant use of other drugs or environmental influences. In fact, in the current study, the concomitant use of more than one substance during pregnancy occurred in 28% of women.

This is also valid for other drugs, such as cocaine and marijuana.^33^ ^34^ We have found a significant alteration in the total Denver scale for cocaine/crack users. In contrast, marijuana use was associated with developmental delay in language and in the total Denver scale. However, the results may have been influenced by concomitant use of other substances, making it difficult to assess the separate impact of these drugs on infant development.

The current study provided new data on substance abuse during pregnancy in Brazilian women and its possible association with some potentially life-threatening conditions in the perinatal period. Furthermore, it shows worrisome data on potential damage to infant development. Among the limitations of the present study, nevertheless, we should highlight that the study design does not allow us to establish a strong association between drug use during pregnancy and unfavorable maternal-fetal outcomes. As a feature of a case-control analysis, information about drug use during pregnancy may have been lost, due to the time elapsed between the childbirth and the interview. Due to the sensitive nature of the topic, there may have been a subnotification of information. Subnotification, however, should be equally balanced between cases and controls.

## Conclusion

The use of psychoactive substances during pregnancy is common. When a composite outcome was considered for the evaluation of maternal morbidity, no association was found between drug use and SMM. However, analysis of diverse causes of morbidity showed that several maternal/perinatal morbid conditions are present in psychoactive substance users during pregnancy. Therefore, it is necessary to broaden the discussion on how to identify, perform follow-up and intervene in cases as well as possible in an effort to reduce the use of psychoactive substances during pregnancy.
